# Durable Response to Chemoimmunotherapy of a Lung Adenocarcinoma Harboring a MET Exon 14 Skipping Mutation

**DOI:** 10.7759/cureus.35545

**Published:** 2023-02-27

**Authors:** Naohiro Nitta, Yoshie Morimoto, Nozomi Tani, Takayuki Shimamoto

**Affiliations:** 1 Department of Pulmonary Medicine, Kyoto Kuramaguchi Medical Center, Kyoto, JPN

**Keywords:** age, adenocarcinoma, non-small cell lung cancer, met exon 14 skipping mutation, chemoimmunotherapy

## Abstract

Chemoimmunotherapy is the first-line standard treatment for patients with non-small cell lung cancer (NSCLC). However, there are few reports on the efficacy of chemoimmunotherapy in patients with NSCLC who harbor the MET exon 14 skipping mutation. We report the case of an 81-year-old male patient with lung adenocarcinoma with a MET exon 14 skipping mutation who was treated with chemoimmunotherapy and achieved a durable response. Chemoimmunotherapy may be a promising treatment option for patients with a MET exon 14 skipping mutation. However, further studies are needed to characterize the objective response rate and response duration in these populations.

## Introduction

A MET exon 14 skipping mutation is a rare oncogenic driver mutation in non-small cell lung cancer (NSCLC) [1]. It accounts for approximately 3% of all NSCLC cases. MET exon 14 includes Y1003, which is the binding site for Casitas B-lineage lymphoma (CBL), an E3 ubiquitin ligase. Hence, when exon 14 is skipped, CBL-mediated degradation of the MET protein is impaired, resulting in excess MET activity [2]. In Japan, two MET inhibitors have recently been approved for patients with advanced NSCLC harboring MET exon 14 skipping mutations [3]. However, clinical trials have shown that more than 50% of these patients experience early relapse within 12 months owing to acquired resistance to these inhibitors [4, 5]. Thus, after the treatment, numerous patients undergo disease progression. In this context, cytotoxic anticancer drugs and immune checkpoint inhibitors (ICIs) are key treatment options for patients with MET exon 14 skipping mutations.

Chemoimmunotherapy has shown to be superior to cytotoxic anticancer drugs and has become one of the standard treatments for NSCLC [6-8]. Moreover, chemoimmunotherapy leads to a lower risk of disease progression compared to ICI monotherapy in patients with NSCLC and oncogenic driver mutations [9]. However, there are few reports on the efficacy of chemoimmunotherapy in patients with NSCLC harboring MET exon 14 skipping mutations. Here, we report the case of a patient with a MET exon 14 skipping mutation who was treated with chemoimmunotherapy and achieved a durable response.

## Case presentation

**Figure 1:**
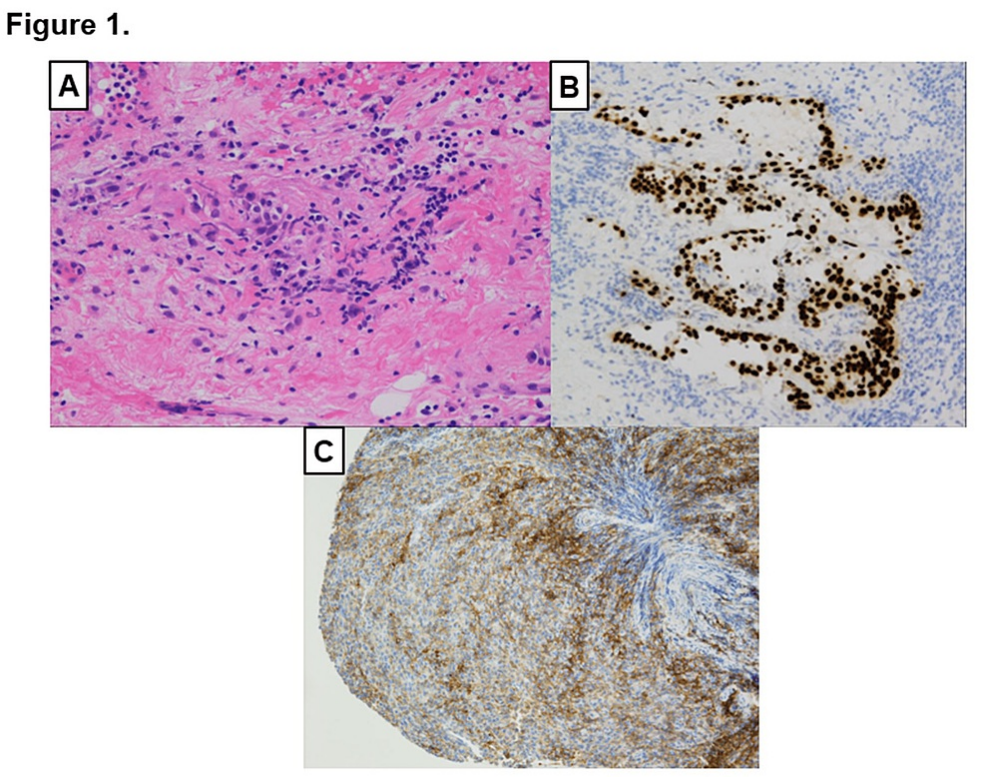
Pathological evaluation of the tissue samples obtained from the right lower lung (A) HE stain (×100). (B) Immunohistochemistry revealed positive TTF-1 (×100). (C) PD-L1 staining using the 22C3 antibody (×100). HE, hematoxylin and eosin; TTF-1, thyroid transcription factor-1; PD-L1, programmed death-ligand 1

An 81-year-old man presented with bloody sputum. He previously smoked 40 cigarettes per day. He had hypertension and old myocardial infarction. The patient’s performance status score was 1. Computed tomography revealed a large right lower lung mass with right adrenal and multiple lymph node metastases. A transbronchial biopsy was performed on the lung mass. Immunohistochemistry revealed positive thyroid transcription factor-1 (TTF-1), Napsin A, and programmed death-ligand 1 (PD-L1) expression (tumor proportion score = 75%) and negative p40 and synaptophysin expression. Therefore, the patient was diagnosed with lung adenocarcinoma (Figure 1)

**Figure 2:**
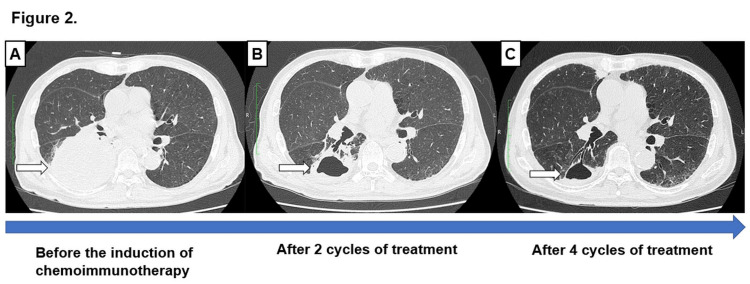
CT axial images (A) CT before the induction of chemoimmunotherapy. (B) CT after two cycles of treatment. (C) CT after four cycles. Remarkable regression of the lower lobe mass lesion of the right lung (arrowhead). CT, computed tomography

Gene expression of epithelial growth factor receptor, anaplastic lymphocyte kinase rearrangement, ROS1 rearrangement, BRAF, and RET fusion was negative in the Oncomine Dx Target Test Multi-CDx system (Pharma Partnerships for NGS in Precision Oncology (oncomine.com)). The patient’s general condition was good; however, because of rapid tumor progression, chemoimmunotherapy was promptly started as the first-line treatment. Carboplatin (area under the curve = 5.0), pemetrexed (500 mg/m^2^dL), and pembrolizumab (200 mg/dL) were administered to the patient every three weeks as the first-line treatment. After four cycles of induction chemotherapy, tumor lesions in the right lower lobe and multiple enlarged lymph nodes almost disappeared, while the tumor lesion in the right adrenal gland was significantly reduced (Figure 2).

Treatment response evaluation indicated that a partial response was achieved. During chemoimmunotherapy treatment, an exon 14 skipping MET mutation was detected by the Oncomine Dx Target Test Multi-CDx system and confirmed by the ArcherMET test (Home-Invitae (archermet.jp)). The Oncomine Dx Target Test Multi-CDx system detected 2739 reads of the MET(13)-MET(15) product. After four courses of induction therapy followed by two courses of maintenance therapy, the patient developed grade 1 pneumonitis. Although the treatment was discontinued, no recurrence was observed for six months. So far, disease control has been achieved up to 11 months after the initiation of chemoimmunotherapy.

## Discussion

Here, we report a case of a patient who received chemoimmunotherapy for lung adenocarcinoma with a MET exon 14 skipping mutation and achieved a durable response. To date, there have been two case reports of patients with the pulmonary sarcomatoid subtype of NSCLC, together with MET exon 14 skipping mutation, who received chemoimmunotherapy [10, 11]. ICIs have shown to be effective in patients with lung sarcomatoid carcinoma [12]. However, to our knowledge, this is the first reported case of advanced lung-not sarcomatoid-adenocarcinoma harboring a MET exon 14 skipping mutation that was treated with chemoimmunotherapy and achieved a durable response.

In this case, chemoimmunotherapy was initiated as the first-line treatment to start treatment as soon as possible. However, in the Japanese Guidelines for Diagnosis and Treatment of Lung Cancer/Malignant Pleural Mesothelioma/Thymic tumor, MET inhibitors are recommended as first-line treatment for patients with MET exon 14 skipping mutation positive lung cancer [13]. Therefore, in actual practice, chemoimmunotherapy is likely to be selected as a second-line treatment. If MET inhibitors are administered as first-line therapy, MET activity becomes immunosuppressive; thus, MET inhibitors may improve the tumor microenvironment, making ICIs effective [14]. Therefore, we consider chemoimmunotherapy to be potentially effective as a second-line treatment.

In our case, the patient developed grade 1 pneumonitis, which improved with treatment discontinuation. His general condition remains good. Careful re-administration of ICIs or MET inhibitors can be performed at the time of relapse. It is known that tumors with MET exon 14 skipping mutations occur more frequently in elderly patients [15, 16], who in turn have a higher frequency of severe adverse events (AEs) and AE-related discontinuations of chemoimmunotherapy [17, 18]. Hence, attention should be paid to the management of AEs during chemoimmunotherapy.

## Conclusions

In conclusion, chemoimmunotherapy may be a potentially effective treatment option for patients with MET exon 14 skipping mutation. Further clinical studies are needed to evaluate the efficacy and safety of chemoimmunotherapy in patients with a MET exon 14 skipping mutation.
